# Two new species of *Dendrophthora* (Viscaceae) from the Venezuelan Andes

**DOI:** 10.3897/phytokeys.140.48865

**Published:** 2020-02-07

**Authors:** Daniela S. Canelón, Santos M. Niño, Laurence J. Dorr, Marcos A. Caraballo-Ortiz

**Affiliations:** 1 BioCentro-UNELLEZ, Herbario PORT, Mesa de Cavacas, Guanare, estado Portuguesa, Venezuela BioCentro-UNELLEZ Guanare Venezuela; 2 Department of Botany, MRC-166, National Museum of Natural History, Smithsonian Institution, P.O. Box 37012, Washington, D.C. 22013–7012, USA National Museum of Natural History, Smithsonian Institution Washington United States of America

**Keywords:** Flora of Venezuela, Guaramacal National Park, Mistletoe, Muérdago, Páramo, Parque Nacional Guaramacal, Subpáramo

## Abstract

Two new species of *Dendrophthora* Eichler (Viscaceae) from northwestern Venezuela are described and illustrated. Both mistletoes, *D.apiculata* Canelón et al., **sp. nov.** and *D.coronata* Canelón et al., **sp. nov.**, are confined to subpáramo and páramo ecosystems of the Venezuelan Andes and are, at present, only known from Guaramacal National Park (Portuguesa and Trujillo states). Ecological aspects and possible taxonomic affinities are discussed. A distribution map also is presented.

## Abstract (es)

Se describen e ilustran dos nuevas especies de *Dendrophthora* Eichler (Viscaceae) del noroeste de Venezuela. Ambos muérdagos, *D.apiculata* Canelón et al., **sp. nov.** y *D.coronata* Canelón et al., **sp. nov.**, se limitan a los ecosistemas subpáramo y páramo de los Andes venezolanos y, en la actualidad, solo se conocen en el Parque Nacional Guaramacal (estados Portuguesa y Trujillo). Se discuten aspectos ecológicos y posibles afinidades taxonómicas. También se presenta un mapa de distribución.

## Introduction

**Figure 1.**
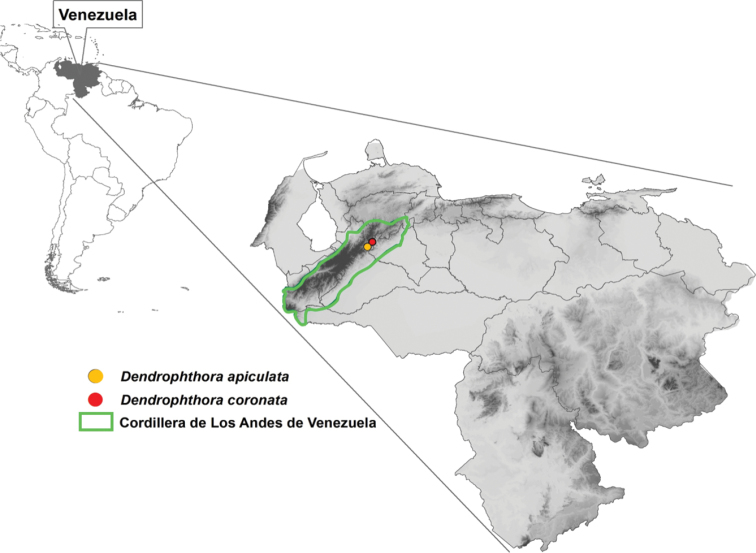
Distribution of *Dendrophthoraapiculata* and *D.coronata* in the Andes of Venezuela. (Source: Centro Cartográfico, UNELLEZ-VPA, 2019).

*Dendrophthora* Eichler (Viscaceae) is the second most diverse genus of mistletoe in the New World comprising over 125 species distributed in Mexico, Central and South America, and the Caribbean (Nickrent in press). We follow Nickrent et al. (2010, 2019) in placing *Dendropthora* in the Viscaceae, even though Stevens (2020) places the genus in a more broadly construed Santalaceae (tribe Visceae Horan.). In South America, the vast majority of *Dendrophthora* species are found at high elevations along the Cordillera de los Andes, ranging from Colombia and Venezuela south to Ecuador, Peru, and Bolivia (Kuijt 1961). Approximately 28 species of *Dendrophthora* have been reported from Venezuela, including 13 endemic species (Rizzini 1982, Kuijt 2008, Tropicos 2019). For Guaramacal National Park, which is located in the northeastern portion of the Venezuelan Andes (Portuguesa and Trujillo states, Fig. 1), Dorr et al. (2000) reported three species of *Dendrophthora*: *D.ambigua* Kuijt, *D.elliptica* (Gardner) Krug & Urb. and an unidentified species. Continued research and collection, focusing on the flora of Guaramacal National Park, revealed an additional unidentified species. Inasmuch as both unnamed species do not match any previously published description of *Dendrophthora* found in the Andes of Venezuela or in the rest of the Americas, we describe them as new and provide information on their habitats and known hosts. Both species apparently are restricted to the subpáramo and páramo ecosystems of the Venezuelan Andes and are, at present, only known from Guaramacal National Park.

## Materials and methods

We studied herbarium specimens of *Dendrophthora* collected in the Venezuelan Andes and deposited in the following herbaria: PORT (Portuguesa, Venezuela) and US (Smithsonian Institution). We also examined the type collection of the latter herbarium, examined specimens in COL (Universidad Nacional de Colombia herbarium, Bogotá, Colombia), and accessed specimens at MO (Missouri Botanical Garden) via the Global Plants JSTOR (2019) and Tropicos (2019) online portals. We also exhaustively reviewed the many articles, monographs, and checklists treating *Dendrophthora* published by Kuijt (1961, 2000, 2003, 2008, 2011, 2016) and our terminology follows his.

Species descriptions were made combining information from fresh and dried specimens, with inflorescences and flowers rehydrated using Aerosol OT solution (Ayensu 1967).

## Taxonomic treatment

### 1. Dendrophthora apiculata

Taxon classificationPlantaeSantalalesSantalaceae

Canelón, S.M.Niño, Dorr & Caraballo sp. nov.

E571F707-DA1C-5F88-9C1B-A90C4636FF91

urn:lsid:ipni.org:names:77205497-1

Figures 2 , 3

#### Type.

Venezuela. Trujillo: Municipio Boconó, Parque Nacional Guaramacal, Páramo de Guaramacal, from summit of Boconó-Caserío de Guaramacal road to the television towers, 2900–3100 m, 13 June 2001, *L.J. Dorr 8953*, with B. Stergios & S.M. Niño (holotype: PORT!; isotypes: K, MO, US-00662868!).

#### Diagnosis.

*Dendrophthoraapiculata* is distinguished from congeners by its rough striate stems; minute, ca. 0.5 mm long cataphylls surrounding all nodes; leaf blades 5–20 × 3–6 mm, apex apiculate with an apiculum 0.2–0.5 mm long; inflorescences usually 1(2) per leaf axil, staminate inflorescences triseriate and pistillate ones uniseriate; flowers ca. 1 mm long; and mature fruits globose-compressed, ca. 0.8–2 × 2–3 mm when dried.

#### Description.

**Figure 2.**
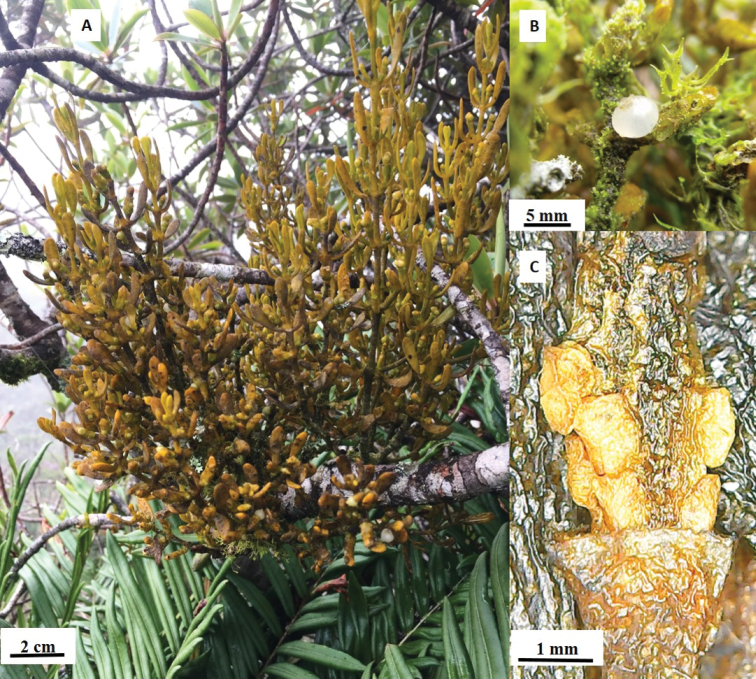
*Dendrophthoraapiculata*. **A** Habit **B** fruit **C** basal flowers (staminate) (Photograph: D. Canelón).

**Figure 3.**
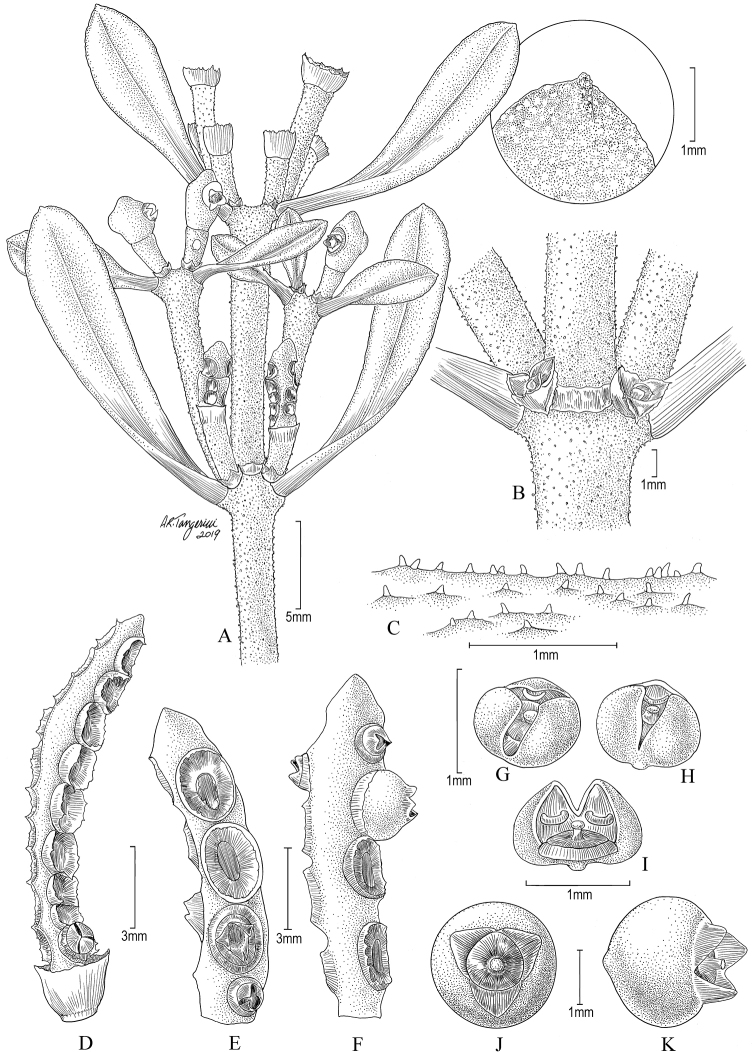
*Dendrophthoraapiculata*. **A** Terminal branches showing inflorescences and leaves, including an enlargement of a leaf apex showing the apiculum (striations are not visible in these terminal branches) **B** Cataphylls at the base of a node **C** Papillose indumentum **D–F** Segments of pistillate inflorescences **G–I** Pistillate flowers **J, K** Mature fruits. (Source: *Stergios et al. 20126*, US).

*Aerial parasitic shrubs*, monoecious; yellowish-green when fresh and drying dark brown. *Stems* woody; erect branches 20–30+ cm long; mature nodes at 2–3 cm long intervals, dichotomous, with multiple branches; coarse, longitudinal striations along principal branches with some transversal striations in basal branches (striations not visible in distal branchlets), minute papillose trichomes dispersed or absent; some lenticels present; cataphylls at nodes ca. 0.5 mm long. *Leaves* opposite, coriaceous; petioles winged, 2–5 mm long, indistinct; blades obovate, 5–20 × 3–6 mm, base cuneate, apex apiculate with an apiculum 0.2–0.5 mm long, margin entire; veins reticulate with midvein evident on the adaxial side when fresh and inconspicuous when dried. Pistillate and staminate *inflorescences* separate, alternate on the same branch or branches either predominantly staminate or predominantly pistillate, usually 1 inflorescence per leaf axil, sometimes 2; fertile internodes usually 1(2), 7–18 mm long; *staminate inflorescences* triseriate; peduncles simple, 1–3 mm long, rugose; cup subtending inflorescence 1.5–1.8 × 2–2.5 mm, edge of cup papillose; basal portion of staminate inflorescences with 9–12 flowers per segment (see Fig. 2C); flowers ca. 1 mm in diameter; embedded in an alveolus (sunken receptacle), emerging up to 2/3 during anthesis; *pistillate inflorescences* uniseriate; peduncles simple, 2–4 mm long, rugose; cup (sensu Kuijt) subtending inflorescence 0.8–1 × 1–2.5 mm, edge of cup papillose, with 3–5 flowers per segment, sometimes more (Fig. 3), flowers adjacent (sometimes touching each other); petals 3(4), triangular, glabrous. *Fruits* globose-compressed, ca. 0.8–2 × 2–3 mm when dried, ripening white-translucent, crowned by persistent petals.

#### Distribution and hosts.

*Dendrophthoraapiculata* is known only from Guaramacal National Park (Trujillo state) between 2600–3100 m on both its northeastern and southwestern mountain slopes. This mistletoe grows in open areas of the páramo and evidently is uncommon throughout its distributional range. Its host range seems to be limited, as the only hosts recorded so far are the shrubs *Cybianthusmarginatus* (Benth.) Pipoly (Primulaceae), *Hypericumjuniperinum* Kunth (Hypericaceae), and *Espeletiagriffinii* Ruiz-Teran & López-Fig. (Asteraceae). Interestingly, the mistletoes found so far on the first host listed were observed on lower and middle branches, while in the last one they were found at the tips of branches.

#### Phenology.

Reproductive individuals of *Dendrophthoraapiculata* have been observed with flowers and fruits all year round, and the fruits seem to be an important source of food for the bird fauna present in the páramo and subpáramo habitats.

#### Etymology.

The specific epithet is derived from “apiculate,” which describes the minute sharp apiculum observed at the apex of leaves.

#### Discussion.

*Dendrophthoraapiculata* is similar to *D.lindeniana* Tiegh., but the latter has stems up to 1 m long, dense papillose trichomes covering the entire plant, leaves with rounded apices with papillose edges, and the cup subtending the inflorescence is usually bifid. In contrast, *D.apiculata* has stems up to 40 cm long, coarsely striate stems with scarce papillose indumentum or papillae absent; leaves with a smooth margin and a persistent, minute apiculum 0.2–0.5 mm long at the apex; and a cup subtending the inflorescence that is usually whole (or rarely rounded).

Regarding their distributions, *Dendrophthoraapiculata* is found in the Páramo de Vicuyal (ca. 2730 m) in Guaramacal National Park (Trujillo state), while *D.lindeniana* grows in the Páramo de Portachuelo (2860 m) (Táchira state), near the border with Colombia. The known localities for these two species are separated one from the other by ca. 240 km.

#### Additional specimens examined.

**Venezuela. Trujillo**: Municipio Boconó, Páramo de Guaramacal, SE of Boconó, 09°10–14'N, 70°11–15'W, 2600–3100 m, 18 July 1990, *L.J. Dorr, L.C. Barnett, W. Diaz, G. Aymard, F. Ortega & N. Murakami 7377* (NY, PORT); Carretera de tierra vía hacia las antenas, 09°14'29.0"N, 70°11'65.0"W, 2800 m, 23 Sep. 2000, *M. Niño, A. Licata & L. Linárez 1385* (US); Sector El Campamento, UTM: 19 368148-1022056 [9.244052N, -70.200324W], 2600 m, 13 Apr. 2019, *S. Niño & D. Canelón 6112* (PORT, US); Páramo de Guaramacal, 3000–3100+ m, July 2002, *B. Stergios & R. Caracas 19754* (PORT, US-00728477); Parque Nacional Guaramacal, Páramo Vicuyal, UTM: 1014040 N, 362685 E [9.171395N, -70.249794W], 2730 m, 11 Apr. 2003, *B. Stergios, L.J. Dorr, S.M. Niño & R. Caracas 20126* (PORT, US-00728399).

### 2. Dendrophthora coronata

Taxon classificationPlantaeSantalalesSantalaceae

Canelón, S.M.Niño, Dorr & Caraballo sp. nov.

F065D133-FA5B-5134-92E5-129C7FD5B9F4

urn:lsid:ipni.org:names:77205498-1

Figure 4

#### Type.

Venezuela. Trujillo: Municipio Boconó, Parque Nacional Guaramacal, trail from antennas on the summit of Páramo de Guaramacal, NE to Fila Los Recostaderos [sic, Recortaderos] (UTM: 19 369344E, 1023140N) [9.253891N, -70.189471W], páramo and subpáramo vegetation, 2677–3100 m, 14 June 2001, *L.J. Dorr 8988* with S.M. Niño & R. Caracas (holotype: US-00662772!; isotype: PORT).

#### Diagnosis.

*Dendrophthoracoronata* is distinguished from congeners by its stems and young branches with longitudinal parallel striations; coroniform trichomes (i.e., papillae crowned by 2–6 minute, simple hairs) covering the entire plant; small, ca. 0.5 × 1 mm cataphylls present at all nodes and sometimes found 1–2 cm above nodes on older branches; petioles 0.5–1 mm long, leaf blades 1–1.5 × 1–1.2 cm, apex rounded and margin slightly crenulate and papillose; uniseriate inflorescences with 5–9 flowers per series; and fruits globose-compressed, 2–3 × 2 mm when mature, white.

#### Description.

**Figure 4.**
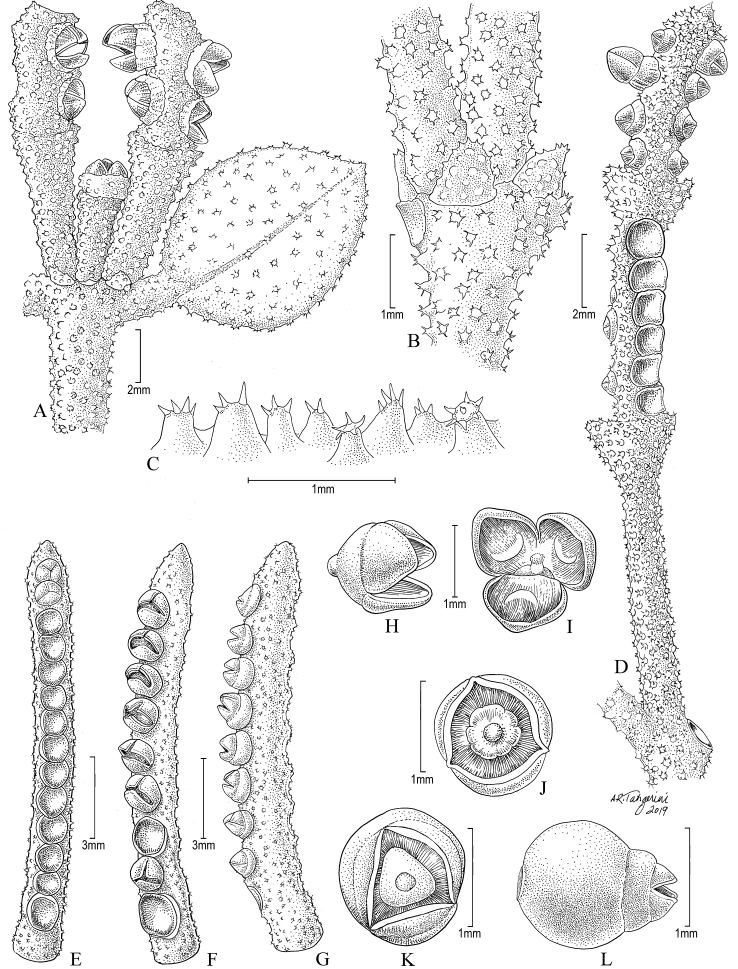
*Dendrophthoracoronata*. **A** Leaf and terminal inflorescences **B** Cataphylls at the base of a node **C** Coroniform trichomes **D** Complete pistillate inflorescence **E–G** Segments of a pistillate inflorescence **H, I** Flowers with vestigial anthers **J–L** Mature fruits. (Source: *Dorr et al. 8988*, US).

*Aerial parasitic shrubs*, monoecious; yellow-orange when fresh, drying blackish or dark green. *Stems* woody, with multiple branches, 30–45 cm long, terete, surface coarse with parallel striations, with a dense layer of coroniform trichomes covering the entire plant; mature nodes separated by 2–3.5 cm long intervals, dichotomous; cataphylls 0.5–1 mm long at nodes, found 1–2 cm above nodes as branches became older. *Leaves* opposite, coriaceous; petioles flattened, 0.5–2 mm long; blades orbicular to elliptic, 1–1.5 × 1–1.2 cm, base slightly cuneate, apex rounded, margin indistinctly crenulate and papillose, surface rough on both sides, veins obscure in dry leaves (Fig. 4). *Inflorescences* completely pistillate within a branch, occasionally with staminate flowers at the base of the inflorescence (completely staminate inflorescences not seen); 1 per leaf axil, 1–3 fertile segments, each 4–10 mm long, uniseriate, greenish when dry; peduncles simple, 6–9 mm long, cup subtending inflorescence 1–1.5 × 3–3.5 mm, almost always forked with a papillate apical edge. *Flowers* 3–9 per segment, 1–1.4 × 0.7–1 mm. *Fruits* globose-compressed, 2–3 × 2 mm, ripening white- or purplish-translucent, surface granulose, tip protruding and crowned with persistent petals.

#### Distribution and habitat.

This species has been found in the transition between cloud forest and subpáramo in Guaramcal. This vegetation is influenced by multiple factors including high rainfall (3200+ mm/year), elevation above sea level (2400–3100 m), as well as relative humidity (100% for most of the year) (Cuello and Cleef 2011). *Dendrophthoracoronata* is found in these Andean/high Andean forests characterized by woody vegetation of low stature with numerous individual shrubs and small trees, and a thin understory with a carpet of thick leaf litter. The canopy can reach between 6–14 m tall, with some emerging trees that reach 16 m, among them: *Ilexguaramacalensis* Cuello & Aymard (Aquifoliaceae), *Miconiajahnii* Pittier (Melastomataceae), *Myrsinedependens* (Ruiz & Pav.) Spreng. (Primulaceae), and *Symplocostamana* Steyerm. (Symplocaceae) (Cuello and Cleef 2011).

#### Phenology.

As with most other species of tropical mistletoe, *Dendrophthoracoronata* can be observed bearing flowers and fruits throughout the year. Its white fleshy fruits seem to be an important food source for forest birds.

#### Etymology.

The species name is derived from the coroniform trichomes (i.e., papillae crowned by 2–6 minute, simple hairs) that cover the entire plant and that resemble small crowns.

#### Discussion.

At first sight, *Dendrophthoracoronata* resembles *D.apiculata* and *D.lindeniana*. However, *D.coronata* can be readily recognized by its marked parallel striations along the stems, small, 0.5–1 mm long basal cataphylls, which sometimes are found 1–2 cm above nodes in old branches, and its dense layer of predominantly coroniform trichomes that cover the entire plant. In contrast, *D.apiculata* and *D.lindeniana* have stems sparsely covered by simple trichomes and lack basal cataphylls (or rarely have a few very small, 0.5 mm long cataphylls and then always at the nodes) (Table 1).

**Table 1.** Morphological characters distinguishing *Dendrophthoraapiculata*, *D.coronata*, and *D.lindeniana*.

	*D.apiculata*	*D.coronata*	*D.lindeniana*
**Plant height**	20–30+ cm.	30–45 cm.	Up to 100 cm.
**Indumentum**	Entire plant sparsely papillate.	Entire plant abundantly covered with coroniform trichomes.	Entire plant abundantly papillate.
**Stem**	With longitudinal striations (not pronounced).	With pronounced striations (furrows).	Without striations.
**Cataphylls**	Cataphylls surrounding nodes 0.2–0.5 mm long; only located at the base of a node.	Cataphylls in basal branches 0.5–1 mm long; found 1–2 cm above a node.	Cataphylls not present.
**Leaf apex**	Apiculate; apiculum 0.2–0.5 mm long.	Not apiculate.	Not apiculate.
**Petiole**	Petiole 2–3 mm long.	Petiole 0.5–2 mm long.	Petiole up to 1 mm long.
**Leaf margin**	Entire.	Slightly crenulate.	Entire.
**Staminate inflorescence**	Triseriate.	Uniseriate.	Uniseriate.

#### Additional specimens examined.

**Venezuela. Trujillo**: Municipio Boconó, Sector El Campamento, UTM: 19 368148 E, 1022056 N [9.244052N, -70.200324W], 2600, 13 Apr. 2019, *S. Niño & D. Canelón 6111* (US); Parque Nacional Guaramacal, sector Vertiente Sur, carretera al caserío Guaramacal, 2000–2750 m, Dec. 1996, *B. Stergios & A. Licata 16813* (US-00656274).

## Supplementary Material

XML Treatment for Dendrophthora apiculata

XML Treatment for Dendrophthora coronata
